# The COVID-19 pandemic impact on continuity of care provision on rare brain diseases and on ataxias, dystonia and PKU. A scoping review

**DOI:** 10.1186/s13023-023-03005-9

**Published:** 2024-02-21

**Authors:** Sara Cannizzo, Vinciane Quoidbach, Paola Giunti, Wolfgang Oertel, Gregory Pastores, Maja Relja, Giuseppe Turchetti

**Affiliations:** 1 Institute of Management, Scuola Superiore Sant’Anna, Pisa, Italy; 2 European Brain Council, Brussels, Belgium; 3 Ataxia Centre, Department of Clinical and Movement Neurosciences, UCL Queen Square Institute of Neurology, London, UK; 4 Philipps-Universitat Marburg, Marburg, Germany; 5 National Centre for inherited Metabolic Disorders, Mater Misericordiae University Hospital, Dublin, Ireland; 6 University of Zagreb Medical School, Zagreb, Croatia; 7 Fulbright Scholar, Institute of Management, Scuola Superiore Sant’Anna, Piazza Martiri della Libertà, 33, 56127 Pisa, Italy

**Keywords:** Rare neurological diseases, Rare neurometabolic diseases, COVID-19 pandemic, Scoping review, Telemedicine, Continuity of care, Discontinuity, Healthcare services delivery, European reference networks, Ataxia, Dystonia, PKU

## Abstract

One of the most relevant challenges for healthcare providers during the COVID– 19 pandemic has been assuring the continuity of care to patients with complex health needs such as people living with rare diseases (RDs). The COVID–19 pandemic accelerated the healthcare sector’s digital transformation agenda. The delivery of telemedicine services instead of many face-to-face procedures has been expanded and, many healthcare services not directly related to COVID-19 treatments shifted online remotely. Many hospitals, specialist centres, patients and families started to use telemedicine because they were forced to. This trend could directly represent a good practice on how care services could be organized and continuity of care could be ensured for patients. If done properly, it could boast improved patient outcomes and become a post COVID-19 major shift in the care paradigm. There is a fragmented stakeholders spectrum, as many questions arise on: how is e-health interacting with ‘traditional’ healthcare providers; about the role of the European Reference Networks (ERNs); if remote care can retain a human touch and stay patient centric. The manuscript is one of the results of the European Brain Council (EBC) Value of Treatment research project on rare brain disorders focusing on progressive ataxias, dystonia and phenylketonuria with the support of Academic Partners and in collaboration with European Reference Networks (ERNs) experts, applying empirical evidence from different European countries. The main purpose of this work is to investigate the impact of the COVID-19 pandemic on the continuity of care for ataxias, dystonia and phenylketonuria (PKU) in Europe. The analysis carried out makes it possible to highlight the critical points encountered and to learn from the best experiences. Here, we propose a scoping review that investigates this topic, focusing on continuity of care and novel methods (e.g., digital approaches) used to reduce the care disruption. This scoping review was designed according to the Preferred Reporting Items for Systematic Reviews and Meta-Analyses extension for scoping reviews (PRISMA-ScR) standards. This work showed that the implementation of telemedicine services was the main measure that healthcare providers (HCPs) put in place and adopted for mitigating the effects of disruption or discontinuity of the healthcare services of people with rare neurological diseases and with neurometabolic disorders in Europe.

**Supplementary Information:**

The online version contains supplementary material available at 10.1186/s13023-023-03005-9.

## Introduction

### Rationale

Coronavirus disease (COVID-19) is an infectious disease caused by the SARS-CoV-2 virus that was first identified at the beginning of 2020 in Wuhan, China. The World Health Organization (WHO) declared the COVID-19 a global pandemic on 11 March 2020. Globally, there have been 773.119.173 confirmed cases of COVID-19, including 6.990.067 deaths, reported to WHO. In Europe there have been 277.962.265 confirmed cases of COVID-19 [1]. Due to the unexpected outbreak of the COVID-19, healthcare systems needed to reorganise the systems concentrating the resources for the care of COVID-19 patients and responding to this health emergency. During the emergency, the discontinuation, the interruption or delay of care, the reorganization of the patients’ care, the respect of the social/physical distancing rules had been some of the main challenges managed by the healthcare organizations including specialist centres/centres of expertise.

COVID-19 pandemic has hardly disrupted the face-to-face health services. Compared with 2019, in May 2020 GP face-to-face consultations decreased by 66% in Portugal, about 18% in Austria and 7% in Norway. The cancer screening and referral were significantly delayed: in Italy − 54% for breast cancer and − 55% for cervical cancer between January and May 2020 compared with the same period in 2019; in Czech Republic in April 2020 -58% screenings for colorectal cancer; in France − 56% for breast cancer screening in the second half of 2020. Elective surgery requiring inpatient stays dropped in many countries in 2020. Emergency visits and admissions in 2020 declined about 28% in Portugal, 21% in UK, about 25% in the Netherlands compared with 2019 [2].

During the emergency, many rare diseases (RDs) patients experienced a very limited access to the diagnostic process and consequently they did not complete their diagnosis pathway; they experienced the interruption of their monitoring care due to the suspension of outpatient and inpatient clinics; they did not access to their healthcare providers because of the impossibility of performing face-to-face evaluations, etc [3].

Telemedicine, the use of information and communication technologies to deliver healthcare at distance, was often proposed as alternative tools to face-face consultations and offered innovative solutions for partially compensating the disruption of the provision of healthcare services and delivering remote care [4, 5]. In Belgium were over 1.2 million teleconsultations in March 2020; in Norway the number of teleconsultations increased from 43,000 in January 2020 to 470,000 in March 2020; in France 4.5 million in April 2020 and 10.9 million teleconsultations performed between January and September 2021. In Germany, there were about 1.4 million video consultations in the first half of 2020 [6].

This scoping review aims at analysing data pertaining to strategies that were implemented for rare neurological diseases and neurometabolic disorders care during COVID-19 pandemic, as well as the impact of these strategies. The present work is part of the results of the European Brain Council (EBC) Value of Treatment (VOT) research project focusing on rare neurological and neurometabolic disorders in Europe. The VoT project looked at early intervention and explored the benefits of coordinated care through the examination of health services, multidisciplinary care patterns (also addressing comorbidity), patient outcomes and costs. The VOT research considered case studies focusing on progressive ataxias, dystonias and phenylketonuria (PKU). Progressive ataxias are a group rare neurological conditions [7] as: hereditary ataxias are a group including Friedreich’s ataxia, spinocerebellar ataxia and episodic ataxia; idiopathic progressive ataxias a group of forms of cerebellar ataxia associated with neurodegeneration of unknown aetiology; specific neurological disorders, in which progressive ataxia is the dominant symptom [8].

Dystonias disorders are still labelled as rare diseases although recent studies stressed that the prevalence has been found increasing. Indeed, estimated prevalence of isolated dystonia in Europe is 16.4/100.000 [9–10].

### Objectives

The main aim of this scoping review was to investigate the impact of COVID-19 pandemic on the continuity of care provision for rare neurological diseases and neurometabolic disorders, and, in particular, Ataxia, Dystonia and Phenylketonuria (PKU) in Europe.

The specific research questions were formulated and defined by consensus among authors using the PCC (Population–Concept–Context) mnemonic [11]. The two research questions were:

1. What have been the changes of the provision of healthcare services for the care of people living with rare neurological diseases (RNDs) and, in particular, with Ataxia, Dystonia and Phenylketonuria (PKU) in Europe during COVID-19 pandemic?
2. What measures (like telemedicine and digital e-tools, etc.) have been implemented for the mitigation of disruption or discontinuity of care for people with RNDs in Europe during COVID-19 pandemic?

In Table 1 is reported the detailed model used for the conceptualization process of the two research questions.

**Table 1:** Scoping review questions

*Scoping review questions*	*Population (P)*	*Concept (C)*	*Context (C)*
1. What have been the changes of the provision of healthcare services for the care of people living with RNDs in Europe during COVID-19 pandemic?	People living with rare neurological diseases and neurometabolic disorders in Europe	Organization of healthcare services provision	COVID-19 pandemic
2. What measures (like telemedicine and digital e-tools, etc.) have been implemented for the mitigation of disruption or discontinuity of care for people with RNDs in Europe during COVID-19 pandemic?	People with RNDs and neurometabolic disorders in Europe	Telemedicine and digital e-tools such as Electronic Health Records (HER), etc.	Disruption or discontinuity of care during COVID-19 pandemic

## Methods

### Protocol and registration

The protocol was drafted following the Preferred Reporting Items for Systematic Reviews and Meta-analysis Protocol - Extension for Scoping Reviews (PRISMA-ScR) [12, 13]. The final protocol was registered on MedRxiv, and it was accessible and available since July 2022 at the following reference: doi: 10.1101/2022.07.26.22277799 [14]. The PRISMA-ScR statement was developed to standardize the greatly heterogenous nature of current scoping review methodology, and comprises a checklist of 22 reporting items, which was developed by an expert panel following recommendations from the Enhancing the QUAlity and Transparency Of health Research (EQUATOR) Network.

The tasks of this review are as follows: (1) defining the key review questions and scope of review, (2) searching and screening studies that meet eligibility criteria based on title and abstracts, (3) identifying relevant studies by screening full texts, (4) extracting and evaluating data, (5) constructing a narrative synthesis summarizing the results.

### Eligibility criteria

To be included in the review the articles must contain a specific field regarding the continuity of care for patients with complex health needs such as people living with rare neurological diseases during the COVID-19 pandemic. In particular, the changes of the provision of healthcare services for the care of people living with Ataxia, Dystonia and PKU in Europe, and the measures (like telemedicine and digital e-tools, etc.) that have been implemented for the mitigation of disruption or discontinuity of care. Moreover, we included all publications examining any dimension of telemedicine such as the synchronous telehealth between health care providers and the patients and families, the monitoring via Apps, smart devices, etc.

Peer-review articles were included if:

- Studies collected data during the COVID-19 pandemic, from January 2020 to the date of our search (September 2022).
- Studies conducted in Europe, written in English, and involving human participants.

Papers were excluded if:

- they did not fit into the conceptual framework of the study, focused on the continuity of care, care pathways, strategies for maintaining provision of care, improving the care provision, and novel methods used to reduce the care disruption in the field of rare neurological diseases, and in particular Ataxia, Dystonia and Phenylketonuria;

c) Were observational or interventional studies, relevant systematic reviews (with or without meta-analysis), relevant scoping reviews;

d) Were editorial articles, pre-print articles, opinion articles and preclinical studies.

### Information sources

Four electronic databases were interrogated: PUBMED, Embase, Scopus and Web of Science from the 1st of January 2020 to the search date (September 2022), using search strings relevant for COVID-19 pandemic, rare neurological diseases and rare neurometabolic disorders, Ataxia, Dystonia and PKU. The search was conducted by the first author and peer reviewed within the research team.

### The search strategy

((COVID-19 or COVID 2019 or severe acute respiratory syndrome coronavirus 2 or 2019 nCoV or SARSCOV2 or 2019nCoV or novel coronavirus) AND (‘rare diseases’ or ‘rare neurological diseases’ or ‘rare neurometabolic disorders’ or Ataxia or Dystonia or PKU or Phenylketonuria) AND (‘care pathway’ or organization or ‘integrated care’ ‘seamless care’ or ‘specialist centres’ or ‘centres of expertise’ or telemedicine or ‘digital health tools’ or continuity or ‘patient care pathway’ or ‘delivery of care’ or economics))

### Screening and data extraction

The search results were exported in Mendeley, and any duplicates removed. Two researchers independently screened titles and abstracts using the literature search results to select papers that meet the inclusion criteria. When necessary, disagreements were managed to reach a consensus. The selected studies were moved to the further step and the full texts were reviewed. The two researchers independently reviewed the full study text and selected the papers that were included in the final review.

## Results

**Fig. 1:**
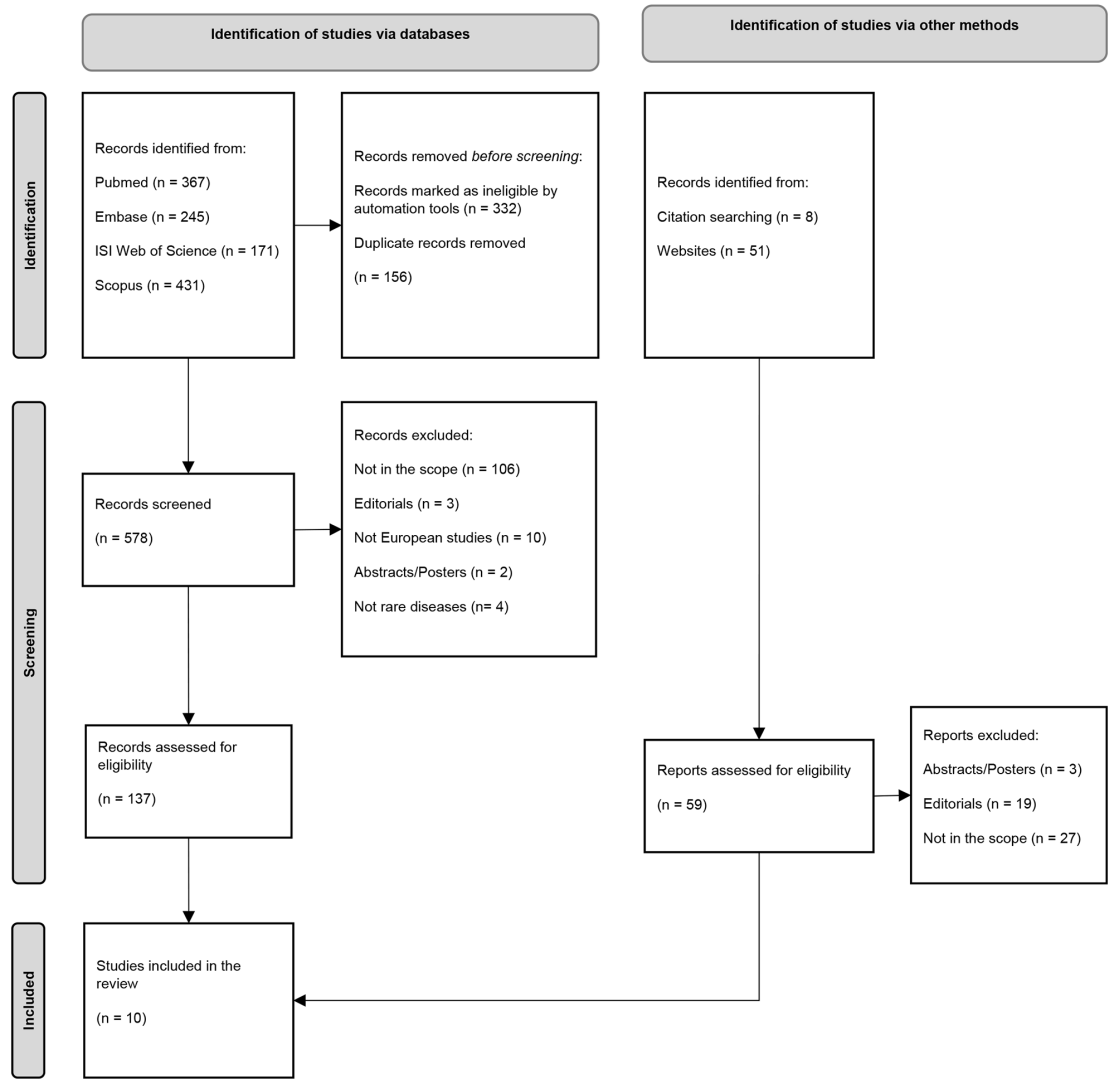
PRISMA flowchart of the results

The total identified studies were 1214 and exported in Mendeley. The tool automatically removed a total of 332 records as unavailable for downloading. After removing 156 duplicates, 578 records were selected for title and abstract screening. The full text screening of n. 137 articles excluded the articles not in the scope of the scoping review, the editorials, the not European studies, the abstracts and the works that do not investigate the rare neurological diseases. The total number of studies included into the scoping review was n. 10. The PRISMA flow chart reported the results of the different phases of the selection process (Fig. 1).

### Data synthesis

A data collection sheet (as separate annex to this work) was developed by the research team to extract studies’ characteristics including citation details (title, author, journal, date, volume, issue, pages), peer-reviewed or grey literature, country/countries involved, aims of the study, methodology, study participant (disease, age, sex, etc.), type of care setting, type of healthcare service provided, the health professionals involved in the treatment(s), the technologies used, key findings and recommendations from the studies.

Most of the ten studies were published during 2021 (n = 7), n = 2 were published in 2022, and only one in 2020. The included articles were from Italy (n = 3), Poland (n = 2), Turkey (n = 2), UK (n = 1), Germany (n = 1), and one study is a European work. A range of publication types were reported: prospective studies (n = 4), retrospective studies (n = 3), surveys (n = 2), and an original article on international expert neurologists’ guidelines.

Two thematic tables are proposed (Tables 2 and 3). Table 2 gives a synthesis of the studies describing the organizational changes adopted in the provision of healthcare services for the care of people living with rare neurological diseases and with neurometabolic disorders in Europe during the COVID-19 pandemic. Table 3 summarises the studies principally focusing on the measures that were implemented during the COVID-19 pandemic for the mitigation of disruption or discontinuity of the care of people living with rare neurological diseases and with neurometabolic disorders in Europe.

**Table 2:** (SR question 1). Studies describing the organisational changes adopted in the provision of healthcare services for the care of people living with rare neurological diseases and with neurometabolic disorders in Europe during the COVID-19 pandemic

Rare Disease(s)	Challenges in healthcare service provision during COVID-19 pandemic	Changes adopted	Results
Data on patients affected by PKU in Italy were collected retrospectively from April 2019 to March 2021 [15]	Follow-up (metabolic management) for both adult and pediatric PKU patients. Before COVID-19 pandemic home monitoring of DBS samplings and on-site visits were performed.	Implementation of slots for video-consultations. Implementation of the composition of the multidisciplinary team (supplemental dietician). Access to the clinic via new service of self-booking online. Activation of tele-visiting: video consultations and long-phone-calls. Activation of a delivery service system directly to the patients’ pharmacies, with the support of the companies that produced medical foods.	Telehealth is more effective in reaching patients who struggle to adhere to the clinic’s face-to-face appointments (working PKU patients).
Data on PKU pediatric patients in Poland collected via a survey in the time of Summer 2020 [16]	Follow-up (metabolic management): communication with doctor/dietician; access to special food. Self-reported blood Phe levels.	Increase of telehealth for contacting the PKU specialist.	Better therapy compliance, more frequent contacts with PKU specialists, and more satisfaction with remote visits.
Rare inherited metabolic disorders and rare autoinflammatory diseases. Data collected by survey. (Adult and pediatric patients in Poland) [17].	Outpatient clinics (ambulatory visits; treatment administration).	Implementation of remote visits as an alternative for regular ambulatory visits (face-to-face).	Despite in Poland most hospital managed to maintain the regularity of visits during the COVID-19 pandemic, wider implementation of remote visits and switch to oral therapy or home infusions would be a good solution to improve patients’ health status.
Cerebellar Ataxia patients. International and European neurologist experts’ guidelines [18].	Closure of ambulatory visits. Ambulatory care services have dramatically decreased.	Virtual video-assisted platforms performing neurology tele-visit (overall mental state, selected eye movements, speech, examination of hyperkinetic movement disorders, ataxia, dexterity, gait). Home-based therapy based on online video tutorials for physical therapy, balance therapy and speech therapy. Behavioral therapists, psychologists, and psychiatrist virtual visits.	Telemedicine has emerged as a principle means of caregiver-patient contact.

**Table 3:** (SR question 2). Studies principally focusing on the measures that were implemented during the COVID-19 pandemic for the mitigation of disruption or discontinuity of the care of people living with rare neurological diseases and with neurometabolic disorders in Europe

Rare Disease	Disruption or discontinuity of the care	Telemedicine/digital tools	Measures implemented	Results
Data on patients affected by Lysosomal disorders (LSD), rare monogenic inherited metabolic disorders were collected from March to June 2020 [19]	Home enzyme replacement treatment ERT infusions interruption (duration of 12 weeks) during the first phase of COVID-19 pandemic. In one year (2020) planned annual review investigations.	Telephone or video consultations.	Remote consultations for outpatient appointments. Remote referrals for 1st appointments; remote transition care. Face-to-face urgent reviews.	Improved patient compliance; reduction of clinic non-attendance; improved collaboration with the GP and local hospitals for regular monitoring. A hybrid model with face-to-face clinics, virtual nurse and doctor-led clinics engagement with local services.
Data of patients with rare neurological diseases, including Ataxia patients were collected from March to September 2020 in Italy [20]	Interruption of planned neurological visits during 2020.	Implementation of a teleconsultation and telemonitoring system.	Televisit performed via individual Teams teleconference connection for each patient follow-up (neurological visits for adults and children, neurosurgical visits, genetic counselling services). Tele-neurorehabilitation delivered to children (RIDInet, with Reading Trainer app; RIDInet with Speech app; Teams). Remote neuropsychological tests for adults. Other telemedicine services provided (clinical multidisciplinary and multidimensional assessment, psychological consultation or support, learning, language and speech rehabilitation, neurofunctional Telemonitoring, and parent coaching).	Telehealth in Italy had limited application in neurological practice until the COVID-19 pandemic mainly because of lack of formal regulations and of recognition as a reimbursable medical service in the NHS. Telehealth was very well received by doctors and also by other healthcare personnel. Telemedicine cold be a valuable tool in particular, for neurological patients needing tertiary neuro-care and living far away from hospital, with motor disability making it difficult to go to hospital, who maintain job activities, or who need frequent monitoring. There are still limitations in the use, for instance the first visits cannot be provided by Telehealth, and the Televisit can be used only for the follow-up of patients with an already diagnosed condition and with well-defined care pathways or needs.
Data on pediatric patients affected by rare neurological diseases, and other complex chronic conditions were collected prospectively in Italy [21].	Ambulatory outpatient visits	Teleconsultations	Design and implementation of an organizational model for telehealth in pediatrics based on three different packages of telemedicine which could be activated according to the medical needs of the patients. Level 1: teleconsultation basic follow-up services performed by videocalls. Level 2: intermediate telemonitoring service including multi-specialist and multi-disciplinary tele-visits; video tutorial dedicated to the parents and caregivers; remote monitoring with specific devices. Level 3: tele-intervention; remote monitoring with specific devices; alert-system active 24/7.	The model facilitated the communication and maintaining standards of care with patients and their caregivers.
Data of Turkey PKU patients retrospectively collected during November 2019/March 2020 vs. March/June 2020 [22].	Interruption of regular outpatient’s clinic services (blood Phe monitoring and assessment of nutritional treatments according to blood Phe levels).	Telemedicine system including: a dedicated e-mail address, teleconference, dedicated phone line; online training meeting on the usage of telemedicine tools.	Online telemedicine platform: phone calls and online communication were recommended to ensure the continuity of follow-up and treatment of PKU patients.	An online and personalized monitoring system can be effective in achieving metabolic control of PKU patients during the COVID-19 pandemic. It has been preferred by both clinicians and patients especially for the follow-up. Finally, telemedicine can facilitate adaptation process and compliance of patients and families to treatment and follow-up in PKU after the pandemic.
Data on patients with movement disorders including Dystonia in Turkey were collected retrospectively from March to June 2020 [23].	Interruption of outpatient services.	Remote communication between the Movement Disorders Unit and the patients by means of phone call, e-mails and chatting via WhatsApp.	Legal barriers in Turkey still exist that do not allow to use video calls with patients.	Despite the limitations, the system allowed clinicians to prevent deterioration of health condition of the patients.
Data on patients with Ataxia in Germany were prospectively collected in 2020 [24].	Impairment in the assessment of Ataxia; interruption of observational and interventional trials.	Video-based system. Performed with any tablet or smartphone and no specific hardware or an examiner needed. A dedicated app was developed to be downloaded by the patients.	Video-based assessment of Ataxia to be done independently of the presence of an examiner applied by the patients themselves at home (Scale for the Assessment and rating of Ataxia - SARA^home^).	SARA^home^ may partly substitute for a conventional SARA assessment in hospitals or research institutions, for example, between scheduled study visits or in situations where the face-to-face visits are not possible to be managed.

## Discussion

**Fig. 2:**
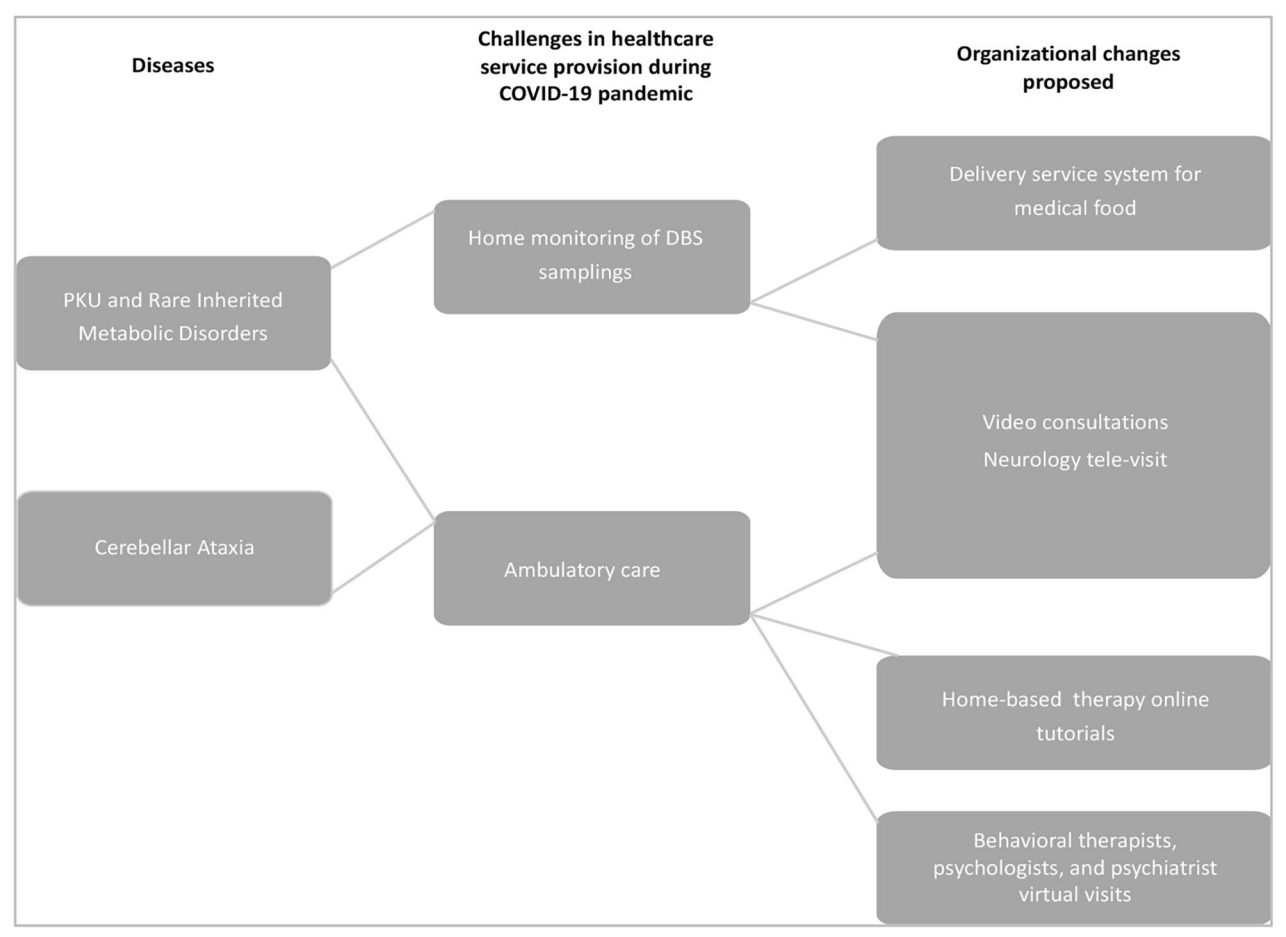
Description of the organizational changes adopted

**Fig. 3:**
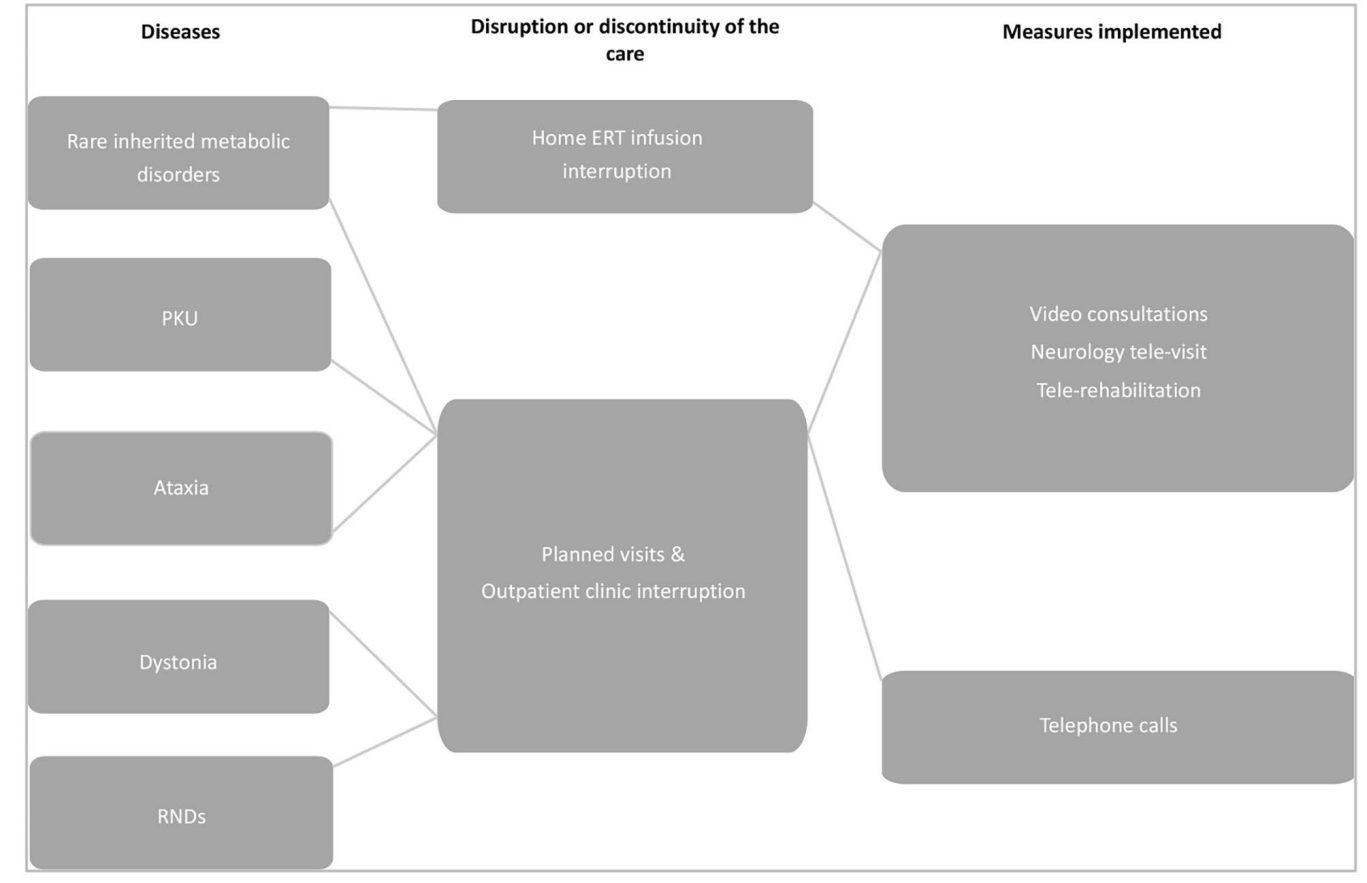
Description of implemented measures implemented

This work is a synthesis of the evidence of changes and strategies aimed at addressing the challenges of disruption of discontinuity of care provision in a very complex healthcare environment represented by the rare neurological diseases and the rare neurometabolic diseases in Europe during the COVID-19 pandemic, focusing in particular on Ataxia, Dystonia and PKU diseases (Figs. 2 and 3).

### Ataxias

The progressive Ataxias are a heterogenous group of rare neurological conditions that typically present with unsteadiness and imbalance, clumsiness, and slurred speech. Patients experience progressive gait problems and in balance difficulties. Dominant Ataxias have an average prevalence of about 2.7:100,000 (range 1.5-4.10 × 10-5), and recessive Ataxias have an average prevalence of about 3.3:1000,000 (range 1.8-4.9 × 10-5) [25–26].

Data on Italian pediatric patients affected by rare neurological diseases, and other complex chronic conditions were collected prospectively in Italy from March to September 2020. The patients experienced the interruption of the planned neurological visits during 2020, and a teleconsultation and telemonitoring system was implemented in order to mitigate the interruption of the care. This remote system implemented televisits that were performed via individual Teams teleconference connection for each patient follow-up (neurological visits for adults and children, neurosurgical visits, genetic counselling services). Tele-neurorehabilitation delivered to children (RIDInet, with Reading Trainer app; RIDInet with Speech app; Teams). Remote neuropsychological tests for adults. Other telemedicine services provided (clinical multidisciplinary and multidimensional assessment, psychological consultation or support, learning, language and speech rehabilitation, neurofunctional Telemonitoring, and parent coaching). Results confirmed that Telehealth was very well received by doctors and also by other healthcare personnel [20]. A German study implemented a pilot video-based instrument, (Scale for the Assessment and rating of Ataxia) SARA^home^, for measuring Ataxia severity at home. The system was developed for the video-based assessment of Ataxia to be done independently of the presence of an examiner applied by the patients themselves at home [24]. Some guidance on how approach the dramatically decrease of ambulatory care services by means of telemedicine in Cerebellar Ataxia was developed by the COVID-19 Cerebellum Task Force. The Author suggested virtual video-assisted platforms performing neurology tele-visit (overall mental state, selected eye movements, speech, examination of hyperkinetic movement disorders, ataxia, dexterity, gait); home-based therapy based on online video tutorials for physical therapy, balance therapy and speech therapy, and behavioral therapists, psychologists, and psychiatrist virtual visits [18].

### Dystonia and other rare neurological diseases

Dystonia syndromes may emerge at any age, and they rarely remit. Virtually any region of the body may be affected, alone or in various combinations. Dystonia may occur in isolation, or it may be combined with other clinical problems. Cervical dystonia is a movement disorder characterized by sustained or intermittent muscle contractions causing patterned and torsional movements, abnormal postures, or both. It has a incidence of about 1.18 × 100.000 person-years [27]. Estimated prevalence rates of adult-onset dystonia in Europe from recent studies range between focal dystonia in Ireland with an estimated prevalence of 17.8 × 10^5^ and all dystonia types in Sweden with a prevalence of 44 × 10^5^ [28]. Variability among epidemiological studies mainly arise from difficulties in ascertaining the diagnosis of dystonias [29].

Data on patients with movement disorders including Dystonia in Turkey were collected retrospectively from March to June 2020. The study aimed at assessing the effects on the implementation of a basic remote communication system mainly based on phone calls, e-mails and chatting via WhatsApp for a partially recovery of the interruption of the outpatients services. Despite the limitations, the system allowed clinicians to prevent deterioration of health condition of the patients [23]. An Italian study on pediatric patients affected by rare neurological diseases, and other complex chronic conditions implemented a comprehensive operational tool including a plurality of services delivered remotely, aimed to cover diagnostic procedures and monitor disease progression in pediatric patients. That organizational model for telehealth in pediatrics was based on three different packages of telemedicine to be activated according to the medical needs of the patients. A first level was a teleconsultation basic follow-up services performed by videocalls. The level 2 was an intermediate telemonitoring service including multi-specialist and multi-disciplinary tele-visits, a video tutorial dedicated to the parents and caregivers, and a remote monitoring with specific devices. The level 3 included also the tele-intervention, the remote monitoring with specific devices and an alert-system active 24/7 [21].

### PKU and rare inherited metabolic disorders

Phenylketonuria (PKU) is an inherited disorder, a rare autosomal recessive inborn error of phenylalanine (Phe) metabolism caused by pathogenic variants in the gene encoding phenylalanine hydroxylase (PAH). PAH deficiency causes abnormal accumulation of Phe in the blood and in the brain. High blood Phe levels are strongly linked to neurocognitive dysfunction, and if not treated PKU causes intellectual disabilities, motor deficits, microcephaly, autism, eczematous rash, seizures, developmental problems, aberrant behavior, and psychiatric symptoms. In Europe PKU prevalence is about 10:100,000 newborns with higher rate in Turkey and Ireland, and a very low rate in Finland. Most of the European countries, the Phe measurement is included in the national newborn screening (NBS) programs [30]. The aim of the NBS is to discover hyperphenylalaninemia (HPA), and this is defined as any blood Phe > 120 µmol/L [31–32].

PKU patients experienced new challenges during COVID-19 pandemic such as the very limited access to the routine care (on-site clinical visits, metabolic controls, blood monitoring frequencies by means of self-sampling), the insecurity regarding the access to the medical and special low-protein foods they need [33]. Despite the movement restrictions and the quarantine measures that many countries implemented during the COVID-19 pandemic, newborn screening and metabolic controls did not experience a discontinuity in especially for children and adolescent patients in Hungary and Italy [34–35]. On the contrary, adherence to the recommended measurement intervals decreased during the pandemic for PKU patients over 16 years and young adults in Austria [36].

Data on patients affected by PKU in Italy collected retrospectively from April 2019 to March 2021 demonstrated that telehealth seemed to be a useful tool to improve the adherence to treatment, it was more effective in reaching patients who made great effort to adhere to the clinic’s face-to-face appointments (such as the working patients) and guaranteed continuous assistance and care during the pandemic. Moreover, PKU patients expressed great satisfaction with the telemedicine offered services [15]. Data collected by surveys showed that caregivers of PKU pediatric patients in Poland reported better therapy compliance, more frequent contacts with specialists during the six-week pandemic period (Summer 2020) and more satisfaction with remote visits than adult patients [16]. Despite in Poland most hospital managed to maintain the regularity of visits during the COVID-19 pandemic, a study focused on patients with rare inherited metabolic disorders and rare autoinflammatory diseases proven that a wider implementation of remote visits and the switch to oral therapy or home infusions would be a good solution to improve patients’ health status [17].

Results on data on patients affected by Lysosomal disorders (LSD) were collected from March to June 2020 in UK where patients experienced the interruption of the home enzyme replacement treatment (ERT) infusions for a duration of 12 weeks during the first phase of COVID-19 pandemic. By means of telephone calls and video consultations the services of remote consultations for outpatient appointments, of remote referrals for first appointments and remote transition care were implemented. The Authors showed improved patient compliance, a reduction of clinic non-attendance, an improved collaboration with the general practitioners (GPs) and the local hospitals for regular monitoring of the patients [19]. In Turkey legal barriers still do not allow the clinicians using video call with patients: a telemedicine system including dedicated e-mail address, teleconference, dedicated phone line, online training meeting on the usage of telemedicine tools was set up for mitigate the impact of interruption or the regular outpatients clinics services on PKU patients. The Turkey study verified that an online and personalized monitoring system can be effective in achieving metabolic control of PKU patients. It was preferred by both clinicians and patients especially for the follow-up [22].

## Conclusions

This work is a synthesis of the evidence of changes and strategies aimed at addressing the challenges of disruption of discontinuity of care provision in a very complex healthcare environment represented by the rare neurological diseases and the rare neurometabolic diseases in Europe during the COVID-19 pandemic, focusing in particular on progressive Ataxias, Dystonias and PKU diseases. Disruption, discontinuity or modification of the care for patients with persistent cerebellar disorders such as ataxia patients negatively influenced the rehabilitation services or the neurological care. Moreover, mental health of these patients may be exposed to be at risk of developing severe complications due to uncertainty, depression and anxiety caused by physical isolation and lack of interaction experienced during the lockdown [37].

This scoping review showed that the implementation of telemedicine services was the main measure that healthcare providers (HCPs) put in place and adopted for mitigating the effects of disruption or discontinuity of the healthcare services of people with rare and neurological diseases and with neurometabolic disorders in Europe. In many countries there are still regulatory, technological, reimbursement, cultural barriers that slow down or impede a wide adoption of these approaches (based on remote technological interaction techniques). Nevertheless, the experience observed during the COVID-19 pandemic as shown that in several circumstances these approaches might be beneficial. If these new patient-healthcare professional-healthcare centre relationship methods might work also “not in emergency conditions” and become routine also in “normal times” will be analysed in the next years.

### Electronic supplementary material

Below is the link to the electronic supplementary material.

Supplementary Material 1

Supplementary Material 2

## Data Availability

The authors declare that the data supporting the findings of this study are available within the paper and its supplementary information files. Extra data are available from the corresponding author upon request.
