# Supplementary material for: The COVID-19 pandemic impact on continuity of care provision on rare brain diseases and on ataxias, dystonia and PKU. A scoping review
Source: Orphanet J Rare Dis. 2024 Feb 21;19:81. doi: 10.1186/s13023-023-03005-9 (PMC10880288; doi:10.1186/s13023-023-03005-9)
Supplement: Supplementary file 1 — Supplementary Material 1 [file 13023_2023_3005_MOESM1_ESM.pdf]

European Brain Council

Brussels, April 13th 2023

The Orphanet Journal of Rare Diseases
Editor in Chief:
Francesc Palau, Hospital Sant Joan de Déu Barcelona and CIBERER, Spain

Dear Professor Francesc Palau,

We are writing to inform you that the European Brain Council (EBC) Value of Treatment research project on rare brain disorders focusing on ataxia, dystonia and phenylketonuria is now coming to an end, and we are aiming to publish study results in the Orphanet Journal of Rare Diseases (OJRD). The study looked at early intervention and explored the benefits of coordinated care through the examination of health services, multidisciplinary care patterns (also addressing comorbidity), patient outcomes and costs. Results provide insight on the value of specialist centres for ataxia and dystonia and metabolic care units for phenylketonuria in terms of diagnosis, management of patients with rare conditions. Case studies which combine a care pathway analysis followed by an economic evaluation were conducted with the support of Academic Partners and in collaboration with European Reference Networks experts, applying empirical evidence from different European countries.
While the idea is to have a joint publication as this would have a greater impact from a public perspective, there should be 6 scientific papers and an EBC Viewpoint. We are submitting them at different stages, as they are ready.

The manuscript we are currently submitting is entitled "The COVID-19 pandemic impact on continuity of care provision on rare brain diseases and on Ataxia, Dystonia and PKU. A scoping review".

The main purpose of this work is to analyze the data related to the strategies that have been implemented for the treatment of rare neurological diseases and neurometabolic disorders during the COVID-19 pandemic, and in particular to investigate the impact of the COVID-19 pandemic on the continuity of care for ataxia, dystonia and phenylketonuria (PKU) in Europe. The analysis carried out makes it possible to highlight the critical points encountered and to learn from the best experiences.

Yours sincerely,

Professor Suzanne Dickson
President

Frédéric Destrebecq
Executive Director

European Brain Council | Rue d'Egmont, 11 | BE - 1000 Brussels
☎ +32 (0) 2 513 27 57 | info@braincouncil.eu | @EU_Brain
V.A.T. : BE 0864 644 340 | www.braincouncil.eu
